# Antioxidant and Antibacterial Activity of *Helichrysum italicum* (Roth) G. Don. from Central Europe

**DOI:** 10.3390/ph15060735

**Published:** 2022-06-10

**Authors:** Zenon Węglarz, Olga Kosakowska, Ewelina Pióro-Jabrucka, Jarosław L. Przybył, Małgorzata Gniewosz, Karolina Kraśniewska, Marek S. Szyndel, Rosaria Costa, Katarzyna Barbara Bączek

**Affiliations:** 1 Department of Vegetable and Medicinal Plants, Institute of Horticultural Sciences, Warsaw University of Life Sciences SGGW, 159 Nowoursynowska Street, 02-776 Warsaw, Poland; zenon_weglarz@sggw.edu.pl (Z.W.); ewelina_pioro_jabrucka@sggw.edu.pl (E.P.-J.); jaroslaw_przybyl@sggw.edu.pl (J.L.P.); 2 Department of Food Biotechnology and Microbiology, Institute of Food Sciences, Warsaw University of Life Sciences SGGW, 159 Nowoursynowska Street, 02-776 Warsaw, Poland; malgorzata_gniewosz@sggw.edu.pl (M.G.); karolina_krasniewska@sggw.edu.pl (K.K.); 3 Department of Plant Protection, Institute of Horticultural Sciences, Warsaw University of Life Sciences SGGW, 159 Nowoursynowska Street, 02-776 Warsaw, Poland; marek_szyndel@sggw.edu.pl; 4 Department of Biomedical, Dental, Morphological and Functional Imaging Sciences, University of Messina, Annunziata Street, 98168 Messina, Italy; rosaria.costa@unime.it

**Keywords:** *Helichrysum italicum*, cultivation, herb, inflorescences, essential oils, phenolic compounds, antioxidant activity, antibacterial activity

## Abstract

*Helichrysum italicum* (Roth) G. Don. is one of the most important cosmetic and medicinal plants originating from the Mediterranean region of Europe. The aim of this study was to assess the chemical profile as well as antioxidant and antibacterial potential of the species cultivated in the temperate climate of Central Europe. The analyses were carried out using herbs and inflorescences. The content of essential oil ranged from 0.25 g × 100 g^−1^ in the herb to 0.31 g × 100 g^−1^ in the inflorescences. Neryl acetate, accompanied by α-pinene in the herb (10.42%), and nerol in inflorescences (15.73%) were the dominants here. Rutoside, as well as rosmarinic, chlorogenic, neochlorogenic, isochlorogenic b and cichoric acids, were detected in both raw materials using HPLC-DAD. Within this group, cichoric acid was the dominant (2647.90 mg × 100 g^−1^ in the herb, 1381.06 mg × 100 g^−1^ in the inflorescences). The herb appeared to be more abundant in phenolics in comparison with the inflorescences. When given antioxidant activity (determined using DPPH and ABTS assays), both methanolic extract and essential oil obtained from the herb indicated higher potential than those originating from the inflorescences (74.72, 61.38 and 63.81, 58.59% in the case of DPPH, respectively). In turn, regarding antimicrobial activity, the essential oil from inflorescences was distinguished by stronger bacteriostatic power than the herb essential oil. Gram-positive bacteria were more sensitive to both essential oils in comparison with Gram-negative ones, with *S. aureus* ATCC 25923 as the most susceptible (MIC 1; MBC 16 mg × mL^−1^) among tested strains.

## 1. Introduction

The genus *Helichrysum* Mill. (Asteraceae family) includes about 600 species distributed all over the world [1,2]. It is represented mainly by xerophytes of bright-yellow flowers which retain their color and form after drying, hence their common name is ‘immortal’ or ‘everlasting’ [3]. *Helichrysum* plants are widely used in traditional and folk medicine [4]. Among them, two species are mostly studied, namely *Helichrysum arenarium* (L.) Moench, growing wild in Europe and Asia, as well as *Helichrysum italicum* (Roth) G. Don. Occurring in the Mediterranean region [5,6,7]. So far, only *H. arenarium* has been officially approved by the European Medicines Agency (EMA) as a herbal medicinal product [8,9]. Nevertheless, due to its specific chemical composition, *H. italicum* has recently drawn special attention and has been recognized as a crop with interesting potential, especially for the cosmetic industry [7,10]. *H. italicum* is a perennial aromatic subshrub, widespread on sunny, rocky slopes and sandy areas from the sea level up to 1700 m. The species consist of several closely related subspecies which easily create hybrids. Thus, it is highly polymorphic both in the morphological and chemical traits [2,11]. The raw materials collected from this plant are inflorescences, and/or aboveground organs (herb), usually hidden under various names, and the so-called: whole plant, aerial part, flower tops, leaves and apical part, flowers and stems, etc. [8,12,13]. Both raw materials are rich in essential oil and phenolic compounds. The total content and chemical composition of essential oils are extremely variable and many chemotypes have been recorded here, with the domination of the following monoterpenes: nerol, neryl acetate, α-pinene, limonene, linalool, geraniol, and sesquiterpenes: γ-curcumene, β-selinene, nerolidol, β-caryophyllene and rosifoliol [14,15,16]. The range of this diversity has already been summarized in review articles by Maksimovic et al. [17], Ninčević et al. [7] and Aćimović et al. [10]. Among phenolic compounds, a lot of chemical subgroups have been identified in *H. italicum,* namely: flavonoids, acetophenones, phloroglucinols, tremetones, coumarins, phenolic acids, and their derivatives [17,18,19,20,21]. In relation to the presence of these secondary metabolites (both volatile and non-volatile), *H. italicum* extracts reveal a wide range of biological activities. The traditional folk medicinal usage includes its application in the treatment of colds, cough, inflammation and liver and gall bladder disorders. Preparations made from this plant, in the form of infusions, decoctions, juices, vapors or powders are administered through ingestion, inhalation or topically [8]. Recently, special attention has been paid to the tissue regeneration and anti-inflammatory effects of the *H. italicum* essential oil, which has resulted in its application in the cosmetic industry as an anti-aging and potentially wound-healing agent in reconstructive surgery [22,23,24]. Both, in vitro and in vivo studies indicate antibacterial activity of the plant, especially against Gram-positive bacteria such as *Staphyloccocus aureus*. However, the data on methicillin-resistant *S. aureus* (MRSA) strains that may cause difficult-to-treat skin infections, are rather scarce [25,26]. When given other biological activities of *H. italicum,* its antiviral, antifungal, antioxidant, and antiproliferative potential has been reported [13,18,27,28,29,30,31,32].

The interest in this plant, especially in the cosmetic industry, has resulted in rising demand for *H. italicum* raw materials, followed by significant exploitation of its wild-growing populations. In turn, the high price of the raw material, especially essential oil, prompted producers to cultivate the plant [10,33,34]. Introducing wild-growing plants into cultivation makes it possible not only to obtain homogeneous, standardized raw material useful for the industry, but also contributes to the protection of natural recourses of such species. In the case of *H. italicum*, this is especially important due to the fact that some habitats where it naturally occurs (e.g., coastal rocks) have already been protected by the European directive [35,36]. According to Ninčević et al. [7], establishing cultivation management followed by breeding research are nowadays the main challenges for this species, considered a new industrial crop. Data concerning these issues are scarce. So far, cultivation trials have been undertaken only in southern European countries, especially the Balkans [16,37,38]. Due to the climatic requirements of the plant, related mainly to high temperature and insolation, the trials to cultivate *H. italicum* in the cooler climate of Central Europe have not been undertaken so far. On the other hand, climate changes clearly observed in recent years, manifested in the region by higher average temperatures in winter and summer, and a longer growing season, are an encouragement to undertake such actions. Taking this into account, the aim of the present study was to determine the yield and quality of herb and inflorescences obtained from *H. italicum* cultivated in the temperate climate of Central Europe, particularly in respect of the antioxidant and antibacterial activity of the raw materials obtained.

## 2. Results and Discussion

Although *H. italicum* provides two different types of raw materials, i.e., herb and inflorescences, in practice they are not always correctly distinguished and are often confused. The proper recognition of the economically used plant organs seems to be crucial not only for its biomass estimation but also in order to determine its biological value based on the chemical composition. For example, in the case of yarrow (*Achillea millefolium* L. s.l, Asteraceae), both herb and inflorescences are collected and used as medicinal products. However, due to the various content of pharmacologically active compounds, their application is different. As a result, the yarrow herb (*Millefolii herba*) has been included in the pharmacopoeial monographs [39] and, when traded for medicinal purposes, it must be chemically standardized. A similar situation can be observed in the case of other members of the Asteraceae family. Regarding this, in the present work, we have assessed the *H. italicum* herb and inflorescences in terms of their yield, chemical composition and biological activity.

### 2.1. Developmental Traits and the Yield of Raw Materials

The results of the study indicate that *H. italicum*, grown in Central Europe climatic conditions, successfully wintered and yielded in the second year of vegetation. The plants created numerous flowering shoots (average 236 per plant), which influenced both the mass of the herb (167.5 g DW per plant) and inflorescences (54.8 g DW per plant). The plants reached about 47.5 cm in height, and their diameter was 92.4 cm (Table 1).

Such results may be related to the advantageous climatic conditions. In Central Europe, the climate can be described as temperate with both continental and maritime elements. It is characterized by relatively cold winters and warm summers, with a strong influence of oceanic air from the west, cold polar air from the east and north and warmer air from the south. The average annual temperature in Poland ranges from 5 to 9 °C, while rainfall reaches up to 700 mm. Long-term changes have been observed over the last several decades: the annual temperature increased by approximately 0.3 °C per decade and the number of days with temperatures below 0 °C decreased by ⅔ days per year. This phenomenon may create new possibilities for crop cultivation on the territory of Poland, especially for species with higher thermal requirements. So far, plants of Mediterranean origin, e.g., common thyme, sage and Greek oregano have been successfully introduced into cultivation. Such attempts with *H. italicum* have not been carried out yet, either in Poland or in other Central European countries. According to Miloradović et al. [40], 3-year-old *H. italicum* cultivated in Serbia was up to 40.76 cm high. The authors claim that 3-year-old plants usually produce about 0.40 kg FW per plant, while 0.45 kg is the yield expected from 4–8-year-old plants.

### 2.2. Essential Oil Content and Composition

In our study, the content of essential oil was higher in the inflorescences (0.31 g × 100 g^−1^) than in the herb (0.25 g × 100 g^−1^) (Table 2). According to the literature data, the content of essential oil in the herb of *H. italicum* may vary from 0.08 to 0.32%, while in the inflorescences from 0.10 to 0.18% [16,41,42,43]. This parameter depends not only on the plant organ (raw material). According to some authors, it is related to the developmental stage of the plant, too [16,41,43].

In the essential oils obtained in our study, 33 compounds were identified, accounting for 94.71% of the total identified fraction in the herb, and 94.75% in the inflorescences. The oxygenated monoterpenes followed by sesquiterpenes hydrocarbons were the main groups in both essential oils. Within monoterpenes, the following compounds were present in higher amounts: neryl acetate, nerol, geraniol, α-pinene, while within sesquiterpenes: α- and β-selinene, italicene, caryophyllene and rosifoliol were identified in higher quantities. The dominant compound both in the herb and in the inflorescences was neryl acetate. So far, at least three main chemotypes have been identified within *H. italicum*, i.e., (I) rich in nerol and its esters, (II) chemotype with a dominance of α-and β-selinene and (III) chemotype with a high amount of γ-curcumene [44]. Taking this into account, *H. italicum* investigated in our study may be classified as the first one. According to Aćimović et al. [10], the chemical diversity of *H. italicum* may be associated with the spatial distribution of its populations. Moreover, since *H. italicum* is regarded as a collective taxon, its chemical variability is enhanced by the morphological one. It is estimated that the species includes several difficult to recognize subspecies, mainly *H. italicum* (Roth) Don ssp. *italicum* and *H. italicum* ssp. *microphyllum* (Willd) Nyman, representing western and eastern Mediterranean groups. This altogether makes the polymorphism of *H. italicum* difficult to describe.

Despite abundant literature data concerning the polymorphic character of the *H. italicum* essential oil, the differences between herb and inflorescences have not been discussed yet. According to our results, the herb essential oil is distinguished by the visibly higher content of neryl acetate (20.27%) and α-pinene (10.42%) and lower content of nerol (4.49%) in comparison with the inflorescences (16.38, 4.05, 15.73%, respectively). Moreover, the share of α- and β-selinene was almost twice as high in the herb than in the inflorescence essential oil (Table 2). When given the biological properties of particular compounds, their percentage share in the essential oil may be crucial from the industrial viewpoint. As mentioned above, in both essential oils analyzed, the dominant compound appeared to be neryl acetate. This compound reveals a number of properties that may be interesting not only to the cosmetic industry but also to the food and the pharmaceutical industry [16,22,45]. In turn, α-pinene shows inhibitory activity on collagenase and elastase enzymes associated with the skin aging process [46]. Both constituents indicate strong antimicrobial activity, too. Thus, the herb essential oil seems to be more adequate to be used in the cosmetic industry than the inflorescence oil.

### 2.3. Content and Composition of Phenolic Compounds

The *H. italicum* raw materials investigated differed according to the total content of phenolic compounds identified (Table 3). The herb appeared to be more abundant in flavonoids (0.19 g × 100 g^−1^) and phenolic acids (1.40 g × 100 g^−1^) in comparison with inflorescences (0.15 and 0.88 g × 100 g^−1^, respectively).

**Figure 1:**
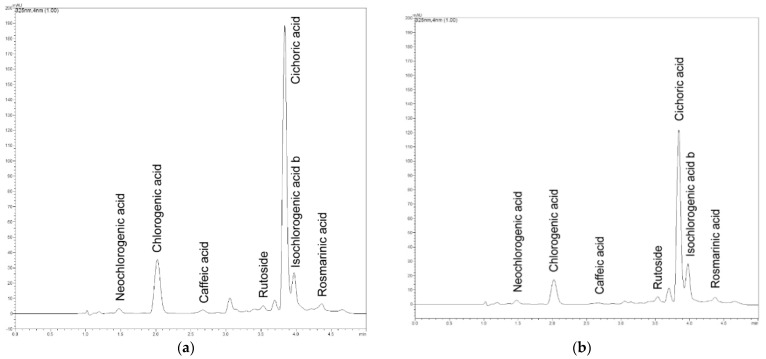
Chromatograms of *H. italicum* herb (**a**) and inflorescences (**b**) methanolic extracts.

**Figure 2:**
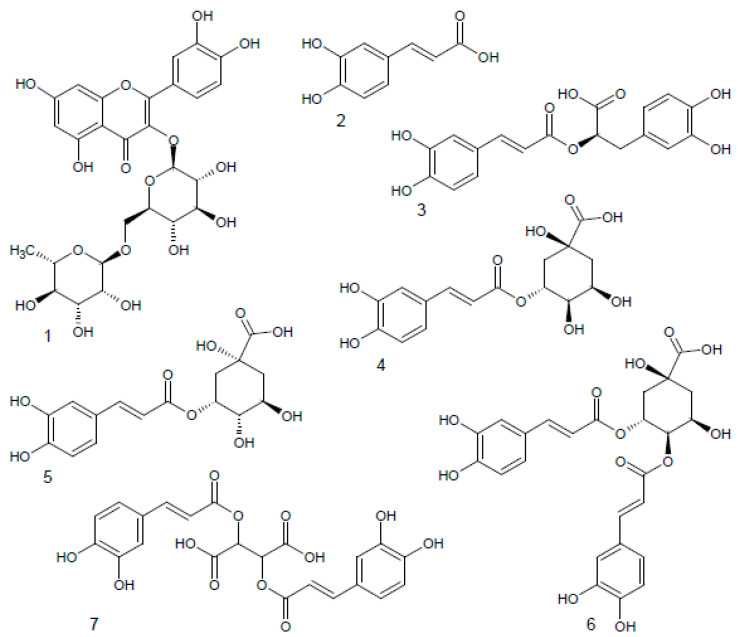
Chemical structures of identified phenolic compounds: (1) rutoside; (2) caffeic acid; (3) rosmarinic acid; (4) chlorogenic acid; (5) neochlorogenic acid; (6) isochlorogenic acid b; (7) cichoric acid.

In both raw materials, seven phenolic compounds were identified using HPLC-DAD. Flavonoids were represented here by rutoside (quercetin-3-O-rutinoside). However, its content was almost eight times higher in the herb than in the inflorescences (191.23 and 24.30 mg × 100 g^−1^) (Table 4, Figure 1 and Figure 2). The results obtained by other authors indicate the presence of other flavonoids in *H. italicum* aboveground organs, including apigenin, isorhamnetin, kaempferol, myricetin, naryngenin, luteolin, galangin, pinocembrin, tiliroside and its derivatives [19,47,48,49]. In our study, six phenolic acids, were identified in the materials analyzed, namely: caffeic, rosmarinic, chlorogenic, neochlorogenic, isochlorogenic b and cichoric acid (Table 4, Figure 1 and Figure 2).

Except for rosmarinic acid, their content was higher in the herb. In both raw materials, cichoric acid was a clear dominant (2647.90 mg × 100 g^−1^, 1381.06 mg × 100 g^−1^, respectively), followed by chlorogenic and isochlorogenic b acids (Table 4). All the acids identified represent cinnamic acid derivatives. Caffeic acid is a free compound, while others are depsides representing its esters. Here, rosmarinic acid is an ester of caffeic and 3,4-dihydroxyphenyllactic acid, while chlorogenic, neochlorogenic, isochlorogenic b and cichoric acids are esters of caffeic and quinic acid, thus belong to caffeoylquinic acid isomers, as following: 3-caffeoylquinic; 5-O-caffeoylquinic; 3,4-di-O-caffeoylquinic and 2,3-dicaffeoylquinic acid. The domination of caffeic acids esters (so-called quinic acid esters) in *H. italicum* raw materials was demonstrated earlier by other authors [17,20,48,49,50]. Kramberger et al. [19] listed hydroxybenzoic and vanillic acid, as well. According to Mari et al. [49], high amounts of quinic acid derivatives may become chemical markers of the species in the standardization procedure.

The third group of phenolics analyzed in the present study were tannins. These substances, similar to other phenolics, are regarded as strong antioxidants and antibacterial agents. Moreover, due to the protein-bonding ability, tannins reveal a strong astringent activity. In our work, it was observed that *H. italicum* inflorescences contained visibly higher content of these compounds when compared to the herb (0.28 and 0.18 g × 100 g^−1^) (Table 3). It is worth noting that the total content of tannins in the herb of yarrow (*Achillea millefolium* L., Asteraceae), known for its strong astringent activity, is at a level of 0.26 g × 100 g^−1^ [51]. So far, the content of these compounds has not been investigated for *H. italicum* raw materials.

### 2.4. Antioxidant Activity

In the present work, the antioxidant activity of both essential oils and methanolic extracts of *H. italicum* herb and inflorescences was determined. It was noticed that the extracts investigated exerted higher activity than essential oils. Regarding raw materials, the herb was characterized by higher antioxidant potential when compared to inflorescences (Table 5) of methanolic extracts, the antioxidant activity may be related to the content of phenolics in the raw materials, especially rutoside and chlorogenic acid, known as strong antioxidants [52,53] (Table 4). Kladar et al. [13] claim that the antioxidant potential of *H. italicum* extract is comparable to quercetin dihydrate, the reference compound used in the food industry in order to prevent the oxidation processes. However, according to Aćimović et al. [10] the activity of *H. italicum* extract is visibly lower than another synthetic antioxidant (butylated hydroxyanisole-BHA). In the case of essential oils, its antioxidant power may be associated with the presence of oxygenated monoterpenes, especially neryl acetate [54,55]. It is worth noting that many world-leading cosmetic companies have developed product lines with the *H. italicum* essential oil, due to its anti-aging effect, which is most likely related to antioxidant properties. So far, the mixture of *H. italicum* essential oil and macerated oil of musk rose was tested on the skin after reconstructive surgery [7,22,56]. According to Andjić et al. [57], *H. italicum* essential oil in formulations such as gel and ointment exhibited a significant wound repairing effect in the incision wound model.

### 2.5. Antibacterial Activity

The minimum inhibitory concentration (MIC) and minimum bactericidal concentration (MBC) values were determined in order to express the antibacterial activity of the *H. italicum* essential oils investigated against selected bacteria, both Gram-negative (*E. coli* ATCC 25922, *P. aeruginosa* ATCC 27853) and Gram-positive (*S. aureus* ATCC 25923, *S. aureus* MRSA ATCC 43300). These strains were chosen for the research due to their pathogenic character, related mainly to soft and subcutaneous tissues, skin and mucous membrane infections [58,59,60,61]. The serial microdilution method has been applied due to its effectiveness and low costs [62]. The essential oils examined indicated different activity on tested strains. In general, Gram-positive bacteria were more sensitive in comparison with Gram-negative ones. Herb essential oil inhibited the growth of *E. coli* and *P. aeruginosa* with MIC values equal to 32 mg × mL^−1^. In turn, inflorescence essential oil showed higher MIC values (64 mg × mL^−1^) which is equivalent to less effective bacteriostatic power. Essential oils from both raw materials did not have bactericidal activity on the above-listed Gram-negative bacteria, in the tested concentration range (MBC ˃ 64 mg × mL^−1^). When given *S. aureus* strains, their growth was more effectively inhibited by inflorescence essential oil (MIC 1–4 mg × mL^−1^) in comparison to the herb one (MIC 4–8 mg × mL^−1^). The bactericidal activity of both essential oils was at a similar level: MBC 16 mg × mL^−1^ towards *S. aureus* ATCC 25923 and 32 mg × mL^−1^ towards *S. aureus* ATCC MRSA 43300. Taking into account MIC and MBC values, the *S. aureus* ATCC 25923 appeared to be more sensitive than *S. aureus* ATCC MRSA 43300, and the most susceptible among all tested strains (Table 6). The differences noticed in the antibacterial activity may be related to the chemical composition of essential oils examined.

The strong antibacterial activity of *H. italicum* essential oils against Gram-positive bacteria was reported earlier by other authors [31,63]. It is considered that such activity is associated with the oxygenated monoterpene content, especially nerol, geraniol and their derivatives [14,64]. This corresponds with the results obtained in our work: inflorescence essential oil contained more above-mentioned compounds than the herb (45.30 and 37.17%, respectively), which was reflected in more effective antibacterial power against both *S. aureus* strains. Here, the limitation of methicillin-resistant *S. aureus* (MRSA) growth should be underlined. This strain has become a serious global problem, since it causes dangerous, hospital-derived infections both internal and external. According to Nostro et al. [25], diethyl ether extract from *H. italicum* inhibits *S. aureus* MRSA by blocking enzymes responsible for its virulence. However, such data on the *H. italicum* essential oil are scarce.

Unlike Gram-positive bacteria, Gram-negative ones are less susceptible to essential oils, including those obtained from *H. italicum*. According to Mollova et al. [34] this essential oil does not show any antibacterial activity against Gram-negative strains. This phenomenon may be correlated to their cell wall structure, which is a complex build using the cytoplasmic membrane, the periplasm and the outer membrane. This altogether acts as a strong permeability barrier and restricts the diffusion of hydrophobic molecules through its lipopolysaccharide covering. Results obtained in our work show that the bacteriostatic activity of *H. italicum* essential oils is twice as strong in the case of herbs than in inflorescences. Such results may be explained by the higher share of α-pinene (10.42%) in this essential oil, which is regarded as a strong antibacterial agent able to destroy cellular integrity, inhibit respiration and ion transport processes [65,66].

## 3. Materials and Methods

### 3.1. Plant Material

The field experiment was carried out at the experimental field of the Department of Vegetable and Medicinal Plants, Warsaw University of Life Sciences (WULS-SGGW). The seeds of *H. italicum* were purchased from Jelitto Staudensamen GmbH (Germany). In February 2018, the seeds were sown in a greenhouse into multi-pots filled with a peat substrate of pH 6.0. In June, the seedlings were planted out into the field on an area of 250 m^2^. The observations of morphological traits and the collection of raw materials were carried out in the second year of vegetation, at the beginning of full blooming (June, 2019). Two raw materials were collected, i.e., herb (upper, non-woody part of shoots with leaves and inflorescences) and inflorescences. Each raw material was collected from 30 randomly selected plants. After the harvest, they were dried at 35 °C, powdered and prepared for the chemical analysis (Section 3.3). A voucher specimen (no 354) was deposited at the herbarium of the Department of Vegetable and Medicinal Plants. Climatic parameters of the 2018 and 2019 vegetation seasons were recorded (Table 7).

Soil parameters (pH, minerals and organic matter content) were measured at the beginning of the second year of plant vegetation by the Agricultural and Chemical Station in Warsaw (Table 8).

### 3.2. Morphological Observations

**Figure 3:**
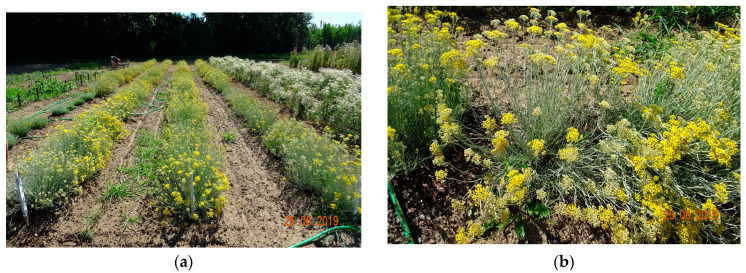
Field experiment overview (**a**) and flowering plants of *H. italicum* (**b**).

**Figure 4:**
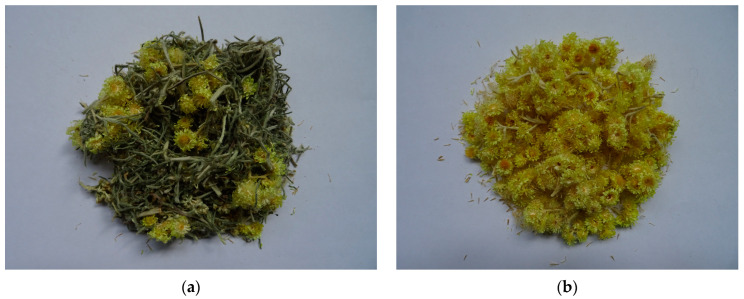
Raw materials from *H. italicum*: herb (**a**), inflorescences (**b**).

Morphological traits were measured directly before the harvest on 30 randomly selected plants, as follows: plant height (cm), number of flowering shoots per plant, plant diameter (cm), fresh (FW) and dry (DW) weight of herb and inflorescences (g per plant). Photographic documentation was performed (Figure 3 and Figure 4).

### 3.3. Chemical Analysis

#### 3.3.1. Essential Oil Content

100 g of air-dried raw material was obtained by 3h hydro-distillation using the Clevenger-type apparatus (Chemland, Warsaw, Poland). The essential oils obtained were stored in dark vials, at 4 °C.

#### 3.3.2. Analysis of Essential Oils by GC-MS and GC-FID

The analyses were carried out by means of an Agilent Technologies 7890A gas chromatograph combined with a flame ionization detector (FID) and MS Agilent Technologies 5975C Inert XL_MSD with Triple Axis Detector (Agilent Technologies, Wilmington, DE, USA). A polar capillary Omegawax^®^ column (30 m × 0.25 mm I.D. × 0.25 µm film thickness) was used. Separation conditions, previously described by Bączek et al. [48], were as follows: oven temperature isotherm at 60 °C for 2 min, temperature rising from 60 °C to 220 °C at a rate of 4 °C per min and held isothermal at 220 °C for 5 min. The carrier gas (He) flow was 1.1 mL × min^−1^. The split ratio was 1:20. Diluted samples (1/100 *v/v*, in n-hexane:isopropanol) of 1 μL were injected at 210 °C by auto sampler. Ion source temperature −220 °C, ionization voltage 70 eV. Mass spectra were scanned in the range of 40–500 amu. Essential oil compounds were identified by matching unknown spectra with those reported in NIST08, NIST27, NIST147, NIST11, Wiley7N2; and on comparison of retention indices (RI) relative to retention times of a series of n-hydrocarbons (C7-C30) with those reported in the literature [67,68]. The percentage composition of the essential oils was computed by the normalization method from the GC peak areas.

#### 3.3.3. Total Content of Phenolic Compounds

The analyses were carried out according to Polish Pharmacopeia 6th [69]. The flavonoid content was determined using the aluminum chloride colorimetric method and expressed as the quercetin equivalent (g × 100 g^−1^ DW), phenolic acids—using Arnov’s method and expressed as caffeic acid equivalent (g × 100 g^−1^ DW), while tannins—using Folin–Ciocâlteu method and expressed as pyrogallol equivalents (g × 100 g^−1^ DW).

#### 3.3.4. Analysis of Phenolic Acids and Flavonoids by HPLC

The air-dried, powdered and homogenized samples (1.000 g) were extracted with 100 mL of methanol for 30 min at 40 °C in a sonication bath (Sonic 6, Polsonic, Warsaw, Poland). The extracts obtained were filtered into amber glass vials with a PTFE 0.22 μm pore and 25 mm diameter syringe tip filter (Sigma-Aldrich, Poznan, Poland).

The Shimadzu Prominence chromatograph equipped with autosampler SIL-20AC HT, photodiode array detector SPD-M20A and LCsolution 1.21 SP1 chromatography software were used (Shimadzu, Kyoto, Japan). The standards were purchased from ChromaDex^®^ (Irvine, Los Angeles, CA, USA) and separately dissolved with methanol in a 25 mL volumetric flask according to the ChromaDex’s Tech Tip 0003: Reference Standard Recovery and Dilution and used as standard stock solutions [70]. Working solutions were set by diluting 10 µL and 100 µL of standard stock solutions with methanol in 10 mL volumetric flasks, 500 µL and 1000 µL in 5 mL volumetric flasks as well as 1000 µL in 2 mL volumetric flasks. Six-point calibration curves were prepared by injecting (1 μL) the working solutions and undiluted stock solution on a column in six replicates (*n* = 6). The curve parameters were estimated in a spreadsheet (Office 365, Microsoft, Redmond, WC, USA) by analyzing the data obtained from the chromatography software. To determine LOD (S/N of 3:1) and LOQ (S/N of 10:1), a signal-to-noise ratio approach was used (Table 9). The separations were achieved on a 100 mm × 4.60 mm C18 reversed-phase column filled with a 2.6 μm solid core with porous outer layer particles (Kinetex^™^, Phenomenex, Torrance, CA, USA) and the binary gradient of deionized water adjusted to pH 2 with phosphoric acid as mobile phase A and ACN as mobile phase B (0.00 min—17% B; 0.50 min—17% B; 3.00 min—40% B; 3.10 min—40% B; 3.10 min—17% B; 5.00 min—stop) with flow rate 2.0 mL × min^−1^, oven temperature 45 °C and injection volume 1 μL. The contents of the compounds determined were calculated as mg per 100 g of dry weight (DW).

### 3.4. Antioxidant Activity

The analyses were performed by DPPH and ABTS scavenging capacity assays using the UV/Vis Shimadzu 1700 PharmaSpec spectrophotometer (Shimadzu, Kyoto, Japan). Raw materials subject to these analyses were previously extracted by methanol at room temperature (10 mL per 1 g raw material) and filtered. Essential oils were obtained using the hydro-distillation method as described above (Section 3.3.1).

#### 3.4.1. DPPH

The measurement was carried out according to Yen and Chen [71] with modifications concerning the time of reaction. Essential oils and extracts were dissolved in methanol. Then, 100 μL of the solution obtained was mixed with 2.9 mL of 0.12 mM DPPH. Absorbance was measured after 30 min at 517 nm. The antioxidant activity of extracts and essential oils was calculated as I = [(AB − AA)/AB] × 100, where I is DPPH inhibition (%); AB is the absorbance of a blank sample (t = 0 min); AA is the absorption of the extract solution (t = 10 min).

#### 3.4.2. ABTS

The analyses were carried out according to the method described by Re et al. [72] and Arts et al. [73]. The stock solution was prepared by stirring 7 mM ABTS and 2.45 mM (final concentration) potassium persulfate in water and incubating at room temperature in the dark, for 16 h before use. The concentrated ABTS was diluted with phosphate-buffered saline (PBS) to the final absorbance of 0.72 (±0.2) at 734 nm. The sample analysis was performed as follows: 1 mL of ABTS solution and 100 μL of sample or standard were mixed. Absorbance of ABTS was measured after 6 min incubation in the dark, at 734 nm. The percentage inhibition of ABTS of the test samples was calculated according to the following formula: % inhibition = [(AB − AA)/AB] × 100, where AB is the absorbance of a blank sample and AA is the absorbance of the test sample.

### 3.5. Antibacterial Activity

Reference strains originated from the American Type Culture Collection (ATCC, Manassas, VA, USA). In the study, two Gram-negative bacteria were used: *Escherichia coli* ATCC 25922 was originally isolated from a human clinical sample and *Pseudomonas aeruginosa* ATCC 27853 which is an opportunistic pathogen. The strains are commonly used in quality control and as controls for antimicrobial susceptibility testing [74,75]. The Gram-positive bacteria: *Staphylococcus aureus* ATCC 25923, commonly used as a quality control strain for commercial products, and methicillin-resistant *S. aureus* (MRSA) ATCC 43300, have drawn our attention due to their multi-resistance to β-lactam antibiotics. MRSA strains have caused a pandemic of mostly skin and soft tissue infections [76].

Frozen bacterial strains were transferred to tryptic soy broth (TSA, BTL, Warsaw, Poland) and incubated overnight at 37 °C in an aerobic condition. Next, the bacterial colonies were harvested and transferred to a sterile 0.85% saline solution (NaCl, POCH, Gliwice, Poland) adjusted to the required optical density of 0.5 on the McFarland turbidity scale (Densimat, bioMérieux, Marcy l’Etoile, France), which is equivalent to a bacterial concentration of 1 × 10^8^ CFU × mL^−1^.

#### Minimum Inhibitory Concentration (MIC) and MINIMUM bactericidal Concentration (MBC)

Minimum inhibitory concentration (MIC) and minimum bactericidal concentration (MBC) were determined using a microdilution assay [77]. A stock solution of essential oils was prepared by dissolving essential oils in a sterile Müller–Hinton Broth (MHB) medium containing 2% of DMSO and 2% of Tween 80. The final stock solution the of essential oil was 64 mg × mL^−1^. To determine the MIC value, a series of twofold dilutions of tested essential oils in the concentration range from 64 to 0.125 mg × mL^−1^ were prepared in MHB, using sterile 96-well microtiter plates (Brand, Wertheim, Germany). Next, to each well on the plates. 10 µL of bacterial inoculum was added, achieving the final concentration of bacteria suspension at around 5 × 10^5^ CFU × mL^−1^. At the same time, two control samples, i.e., negative (medium and inoculum without essential oils) and positive (medium and essential oils without inoculum) were prepared. The microtiter plates were incubated at 37 °C for 24 h. The MIC value was defined as the lowest concentration of essential oils that completely inhibited bacterial growth.

MBC was determined by sub-culturing 10 µL from each well with no bacterial growth to plates with the Müller–Hinton Agar medium. The plates were incubated at 37 °C for 24 h. The MBC was defined as the lowest concentration of essential oil killing 99.9% of the bacterial inocula.

### 3.6. Statistical Analysis

Data were subjected to a statistical analysis using the Statistica 12 software (Krakow, Poland). The mean values were compared using the one-way analysis of variance (ANOVA) and expressed as mean with standard deviation (±SD). The differences between individual means were signed as ‘*’ in table rows and considered to be significant at *p* < 0.05.

## 4. Conclusions

The obtained results indicate that *H. italicum* can be successfully cultivated in the temperate climate of Central Europe. High yields of the herb or inflorescences, already in the second year of vegetation of this perennial, can be obtained. In the present study, these raw materials have been comprehensively compared with respect to the chemical profile of their essential oils and phenolics, followed by their antioxidant and antimicrobial activity. It was shown that the inflorescences were characterized by a high content of essential oils and tannins while the herb—of flavonoids and phenolic acids. In the essential oil from the inflorescences, the dominants were neryl acetate and nerol while from the herb neryl acetate and α-pinene. In both raw materials, the contents of eight phenolic compounds were detected, with cichoric acid as a dominant. Its content was twice as high in the herb as in the inflorescences. Regarding biological activity, extracts from the herb indicated higher antioxidant potential, but bacteriostatic power was higher in the case of inflorescences. The above-mentioned differences noticed between *H. italicum* raw materials should be taken under consideration when their industrial application is concerned. Obtained results may be developed in future investigations on *H. italicum* regarding both its cultivation aspects and usage in phytotherapy as an antimicrobial agent.

## Figures and Tables

**Table 1:** Morphological traits and the mass of the plants.

Investigated Traits	
Plant height (cm)	47.5 ± 3.9
Number of flowering shoots per plant	236.1 ± 31.2
Plant diameter (cm)	92.4 ± 8.1
Fresh weight of herb (g × plant^−1^)	438.5 ± 32.3
Dry weight of herb (g × plant^−1^)	167.5 ± 30.4
Fresh weight of inflorescences (g × plant^−1^)	141.3 ± 28.4
Dry weight of inflorescences (g × plant^−1^)	54.8 ± 6.4

**Table 2:** The content (g × 100 g^−1^) and chemical composition (% area) of essential oils.

No	Compound	RI ^a^	RI ^b^ Range	Herb	Inflorescences
	Essential oil content			0.25	0.31
1	α-pinene	1029	1008–1039	10.42	4.05
2	camphene	1074	1043–1086	0.28	0.06
3	β-pinene	1112	1085–1130	0.10	0.08
4	δ-3-carene	1150	1122–1169	0.18	0.25
5	α-terpinene	1185	1154–1195	0.06	0.12
6	limonene	1204	1178–1219	2.17	0.82
7	eucalyptol	1214	1186–1231	0.30	0.82
8	p-cymene	1275	1246–1291	0.40	0.44
9	terpinolene	1284	1361–1300	0.19	0.15
10	3-methyl-2-butenoic acid	-	-	0.16	0.55
11	α-copaene	1495	1462–1522	0.16	1.01
12	β-cubebene	1538	1518–1560	1.87	1.12
13	linalool	1542	1507–1564	0.49	2.25
14	cis-α-bergamotene	1559	1534–1580	0.38	1.03
15	(E)-caryophyllene	1594	1570–1685	4.06	4.50
16	α-humulene	1659	1637–1689	0.00	0.09
17	italicene	-	-	6.89	7.25
18	neral	1678	1641–1706	2.42	1.91
19	β-selinene	1711	1686–1743	8.20	4.63
20	neryl acetate	1721	1693–1740	20.27	16.38
21	α-selinene	1726	1696–1748	9.05	5.27
22	ar curcumene	1775	1743–1788	3.43	3.82
23	α-cadinene	1794	1734–1803	0.00	0.84
24	nerol	1797	1752–1832	4.49	15.73
25	geraniol	1814	1795–1865	6.80	6.32
26	guaiol	2088	2061–2104	1.07	1.40
27	rosifoliol	-	-	3.82	6.40
28	humulane-1.6-dien-3-ol	-	-	0.70	0.52
29	α-eudesmol	2221	2186–2250	1.13	1.08
30	cubenol	2063	2026–2090	0.00	1.62
31	β-eudesmol	2235	2196–2272	0.37	0.50
32	carvacrol	2210	2140–2246	2.41	1.88
33	selinen-4-α-ol	2251	2207–2274	2.44	1.86
	Total:			94.71	94.75
	Monoterpene hydrocarbons			13.40	5.53
	Oxygenated monoterpenes			34.77	43.41
	Sesquiterpene hydrocarbons			34.04	29.56
	Oxygenated sesquiterpenes			9.53	13.38
	Others			2.97	2.87

^a^ RI—experimental retention index on polar Omegawax^®^ column. ^b^ RI—range of retention indexes on polar column reported in the literature (Refer to Section 2.5 for more details).

**Table 3:** The total content of phenolics (g × 100 g^−1^ DW).

No	Group of Compounds	Herb	Inflorescences
1	Flavonoids	0.19 ± 0.02 *	0.15 ± 0.01
2	Phenolic acids	1.40 ± 0.11 *	0.88 ± 0.05
3	Tannins	0.18 ± 0.01	0.28 ± 0.02 *

* *p* < 0.05.

**Table 4:** The content of detected phenolic compounds (mg × 100 g^−1^ DW).

No	Compound	Herb	Inflorescences
Flavonoids			
1	Rutoside	191.23 ± 6.50 *	24.30 ± 12.99
Phenolic acids			
2	Caffeic acid	41.71 ± 1.17 *	27.26 ± 1.15
3	Rosmarinic acid	53.39 ± 7.76	64.48 ± 7.87
4	Chlorogenic acid	338.81 ± 7.32 *	142.94 ± 6.56
5	Neochlorogenic acid	85.83 ± 3.38	59.41 ± 1.22
6	Isochlorogenic acid b	435.10 ± 7.49 *	162.61 ± 6.58
7	Cichoric acid	2647.90 ± 62.20 *	1381.06 ± 31.77
	Sum	3793.96 *	1862.04

* *p* < 0.05.

**Table 5:** Antioxidant activity of essential oils and methanolic extracts (% RSC).

Method	Essential Oils		Methanolic Extracts	
	Herb	Inflorescences	Herb	Inflorescences
DPPH	61.38 ± 0.54	58.59 ± 0.73	74.72 ± 0.77	63.81 ± 0.30
ABTS	67.78 ± 0.31	60.53 ± 0.65	81.96 ± 0.38	72.48 ± 0.41

**Table 6:** MIC and MBC values of essential oils (mg × mL^−1^).

Bacteria Strains	Herb		Inflorescences	
	MIC	MBC	MIC	MBC
*E. coli* ATCC 25922	32	˃64	64	˃64
*P. aeruginosa* ATCC 27853	32	˃64	64	˃64
*S. aureus* ATCC 25923	4	16	1	16
*S. aureus* MRSA ATCC 43300	8	32	4	32

**Table 7:** Climatic parameters.

Month	Year	Min. Temperature (°C)	Max. Temperature (°C)	Rainfall (mm)	Air Humidity (%)	Sun Days	Sun Hours
January	2018	−1	2	35.5	80	11	191
	2019	−4	0	65.1	85	7	137
February	2018	−4	−1	18.5	74	13	156
	2019	0	5	27.3	80	14	218
March	2018	−2	4	29.8	74	10	173
	2019	3	10	42.1	68	8	239
April	2018	8	19	26.9	67	14	315
	2019	6	16	26.3	59	19	318
May	2018	12	23	110.7	68	11	350
	2019	9	18	14.6	74	4	275
June	2018	13	24	72.2	65	5	327
	2019	16	27	73.0	70	9	357
July	2018	16	26	165.5	70	3	344
	2019	13	22	102.9	74	3	314
August	2018	16	27	56.1	63	14	349
	2019	15	25	66.2	66	8	352
September	2018	12	22	67.4	68	17	323
	2019	11	19	78.5	71	12	274
October	2018	8	16	50.1	68	20	292
	2019	9	16	31.4	73	18	316
November	2018	3	8	19.3	77	21	280
	2019	4	9	34.8	78	16	269
December	2018	−1	3	73.5	86	5	131
	2019	2	5	48.3	80	10	188

**Table 8:** Soil parameters.

pH	NO_3_^−^ (mg × L^−1^)	NH_4_^+^ (mg × L^−1^)	P_2_O_5_ (mg × 100 g^−1^)	K_2_O (mg × 100 g^−1^)	Mg (mg × 100 g^−1^)	Organic Matter (%)
6.05	75	23	21.9	95.0	21.9	2.71

**Table 9:** HPLC-DAD validation parameters (*n* = 6).

No.	Compound	Precision Intra-Day (CV %)	Precision Inter-Day (CV %)	Calibration Equation	R^2^ (*n* = 6)	Linear Range (mg × mL^−1^)	LOD (µg × L^−1^)	LOQ (µg × L^−1^)
1	Rutoside	0.37	0.86	*y* = 1434.0*x* − 5093.0	0.9999	0.90–90.67	74.6	248.8
2	Caffeic acid	1.00	1.72	*y* = 2592.9*x* + 379.6	0.9996	1.00–998.40	2.50	8.32
3	Rosmarinic acid	1.24	2.12	*y* = 2017.9*x* + 1100.4	0.9999	0.43–434.02	3.20	9.82
4	Chlorogenic acid	1.32	1.63	*y* = 6517.4*x* − 12016.6	0.9997	0.40–39.47	20.97	69.90
5	Neochlorogenic acid	0.27	0.78	*y* = 1809.0*x* − 1539.8	0.9999	0.39–392.0	18.39	61.31
6	Isochlorogenic acid b	0.81	1.23	*y* = 3782.2*x* − 4613.2	0.9994	0.19–190.00	9.56	31.87
7	Cichoric acid	0.18	0.49	*y* = 3230.70*x* + 6882.20	0.9998	0.46–456.96	11.47	38.23

## Data Availability

All data is contained within article.
